# Threat perception and familiarity moderate the androgen response to competition in women

**DOI:** 10.3389/fpsyg.2013.00389

**Published:** 2013-07-05

**Authors:** Gonçalo A. Oliveira, Sara Uceda, Tânia Oliveira, Alexandre Fernandes, Teresa Garcia-Marques, Rui F. Oliveira

**Affiliations:** ^1^Unidade de Investigação em Eco-Etologia, Instituto Superior de Psicologia Aplicada – Instituto UniversitárioLisbon, Portugal; ^2^Laboratorio de Psicobiologia, Departamento de Psicologia Experimental, Universidad de SevillaSeville, Spain; ^3^Unidade de Investigação em Psicologia Cognitiva, do Desenvolvimento e da Educação, Instituto Superior de Psicologia Aplicada – Instituto UniversitárioLisbon, Portugal; ^4^Champalimaud Neuroscience Programme, Instituto Gulbenkian de CiênciaOeiras, Portugal

**Keywords:** cognitive appraisal, threat, challenge, familiarity, testosterone, competition

## Abstract

Social interactions elicit androgen responses whose function has been posited to be the adjustment of androgen-dependent behaviors to social context. The activation of this androgen response is known to be mediated and moderated by psychological factors. In this study we tested the hypothesis that the testosterone (T) changes after a competition are not simply related to its outcome, but rather to the way the subject evaluates the event. In particular we tested two evaluative dimensions of a social interaction: familiarity with the opponent and the subjective evaluation of the outcome as threat or challenge. Challenge/threat occurs in goal relevant situations and represent different motivational states arising from the individuals’ subjective evaluation of the interplay between the task demands and coping resources possessed. For challenge the coping resources exceed the task demands, while threat represents a state where coping resources are insufficient to meet the task demands. In this experiment women competed in pairs, against a same sex opponent using the number tracking test as a competitive task. Losers appraised the competition outcome as more threatening than winners, and displayed higher post-competition T levels than winners. No differences were found either for cortisol (C) or for dehydroepiandrosterone. Threat, familiarity with the opponent and T response were associated only in the loser condition. Moderation analysis suggests that for the women that lost the competition the effect of threat on T is moderated by familiarity with the opponent.

## INTRODUCTION

The responsiveness of androgens to social interactions has been established in behavioral endocrinology for many years (Wingfield et al., 1990; Oliveira, 2004). Testosterone (T) is known to respond in anticipation to a social challenge and as a function of its outcome, and this response is moderated by social context (Hsu et al., 2006; Oliveira, 2009). According to the “challenge hypothesis” (Wingfield et al., 1990), these changes in T levels have the function of adjusting the expression of T-dependent aggressive behavior to social context, thus avoiding the costs associated with keeping chronically elevated T levels when no social challenges are present or anticipated. The challenge hypothesis was originally proposed in birds to explain inter-specific variation in androgen response to social challenges (Wingfield et al., 1990) and it has been subsequently extended to other taxa from invertebrates to humans (Archer, 2006; Hirschenhauser and Oliveira, 2006; Scott, 2006).

Although most studies investigating the androgen response to social interactions have focused on T, recent studies have shown that in species that exhibit aggressive behavior outside the breeding season, when gonadal steroids are low, adrenal androgens such as dehydroepiandrosterone (DHEA) may also regulate aggressive behavior (Soma and Wingfield, 2001; Soma et al., 2008). In humans, DHEA is also a major circulating androgen, mostly produced in the adrenal cortex and has known effects on aggressive behavior, emotion processing, and cognitive functions (Wolf and Kirschbaum, 1999; Soma et al., 2008). A negative correlation between changes in DHEA and avoidance behavior has been reported (Rasmusson et al., 2004) and adolescent girls with aggressive conduct disorders show lower cortisol to DHEA ratios when compared with girls with non-aggressive conduct disorder (Pajer et al., 2006).

Despite the findings that in women, T predicts the reaction to winning or losing (Josephs et al., 2006; Mehta et al., 2008) and that this androgen is associated with status and dominance (Grant and France, 2001; Cashdan, 2003; Edwards et al., 2006; Wirth and Schultheiss, 2007), only recently a winner/loser effect in the direction predicted by the challenge hypothesis was reported in a female soccer competition (Oliveira et al., 2009). Further evidence for the relevance of investigating the responsiveness of T in competing women was recently provided by Jiménez et al. (2012), who showed that men and women present the same pattern of T variation (a winning/losing effect) in response to a competitive event. Previous research had shown post-competitive increases of T both in winners and losers (e.g., Bateup et al., 2002; Edwards et al., 2006; Edwards and O’Neal, 2009; Hamilton et al., 2009). These contradictory results can be due to a modulatory role of psychological variables that have not been accounted for in previous studies. For instance, van der Meij et al. (2010) in an all male sample found a post-competitive increase of T for both winner and losers that was moderated by opponent self-efficacy (i.e., higher T response when the opponent had higher self-efficacy). Thus, the within-species variation in the androgen responsiveness to competition that has been documented both in females and males across different studies may be due to a moderator role of conditional and contextual variables (Archer, 2006; Salvador and Costa, 2009). This view has prompted the quest for the identification of psychological moderator and mediator variables between competition and androgen responsiveness in human research, where sports competition or vicarious competition laboratory tasks are used as proxy for dominance contests (for reviews see Salvador, 2005; Archer, 2006; van Anders and Watson, 2006; Salvador and Costa, 2009; Carré et al., 2011). Personality traits (e.g., implicit power motivation and coping styles) and affective and cognitive variables (e.g., causal attribution, mood, and perceived self-efficacy of the opponent) have been shown to have an effect on the androgen response (Salvador, 2005; Salvador and Costa, 2009; Stanton and Schultheiss, 2009; van der Meij et al., 2010).

One key set of moderator variables of the androgen response is the cognitive appraisal of the competition (i.e., the significance of competition to the individual), such that rather than the objective structure of the competition it is the subject’s perception of the event that triggers the androgen response (Oliveira et al. , 2005). Within this hypothesis, psychological variables that are central for the appraisal of the competition consequences to the subject, such as perception of the outcome as threat vs. challenge and the familiarity with the opponent, have not been investigated in humans so far. Although rooted in the classic appraisal theory (Lazarus, 1991; Scherer, 2001) the processes investigated here are less conscious and more automatic than the ones usually labeled as “cognitive appraisal” in the appraisal psychology literature. Therefore, following Blascovich (2008) hereafter we will use the term “evaluation” to refer to these processes.

Challenge and threat represent person-situation evoked motivational states, that can drive behavior and increase performance, involving the interplay of affective (feelings and emotions) and cognitive processes (attention and appraisal). Challenge and threat occur in goal relevant situations, they present different patterns of psychological and physiological response, and depend of the balance between the event demands and the perceived coping capacity of the individual (Tomaka et al., 1993,^1997^; Blascovich and Mendes, 2000). Evaluation of an event as a threat can occur when the resources of the individual are insufficient to meet the demands (Tomaka et al., 1993,^1997^). Individuals with a threat evaluation report higher subjective stress and display lower cardiac reactivity (i.e., heart rate, pre-ejection period and cardiac output) and increased vascular resistance (i.e., vasoconstriction). The evaluation of an event as challenge appears when the individual experiences sufficient resources to meet the event demands (Tomaka et al., 1993,^1997^). There is lower subjective stress when compared to the threat response and it is accompanied by high cardiac reactivity and low vascular resistance, which have been interpreted as a marker of the individual effort to cope with the task demands and mobilize resources to remain in control of the situation (Tomaka et al., 1993,^1997^). There is also some evidence that the physiological response is not causally antecedent to the evaluation reported by the individual, as the manipulation of the specific pattern of physiological activity of threat and challenge did not produce an evaluation of a stressor consistent with the physiological activation (Tomaka et al., 1997). Furthermore, since appraisal is also a continuous evaluation process that is updated by the constant flow of information that the organism receives from the environment, the appraisal process implies a subjective selection of relevant information to serve as a basis for the evaluation of the event (Scherer, 2001). Together appraisal theory suggests that the evaluation depends more on how it is experienced by the individual than on the event itself. Specifically in this experiment, we have investigated how winning and losing is evaluated by the participants and in what manner that evaluation of the outcome may affect the endocrine response to competition. Given that familiarity serves as a primary criteria for the selection of relevant information in the appraisal process (Scherer, 2001), this variable was also accounted for in our experiment.

The effects of familiarity on competition have been extensively studied in animals, where the aggressive response depends on the relative threat posed by familiar vs. stranger opponents. In social systems with aggregated stable territories territorial neighbors (familiar opponent) pose less threat than floaters (unfamiliar opponent) that could be looking for a territorial take-over and therefore and elicit less aggression (“dear enemy effect,” e.g., Ydenberg et al., 1988; Temeles, 1994). There is also some evidence that in other species, familiar opponents heighten the aggressive response. In these groups, neighbors pose a more significant threat for territorial usurpation or mating competition than roaming strangers that are commonly outnumbered by their same sex rivals in the established social groups (Muller and Manser, 2007). A pilot study in our lab has shown that in cichlid fish territorial intrusions by a familiar opponent elicit lower androgen responses than intrusions by strangers (R. F. Oliveira, R. Aires, T. Oliveira, and A. Ros, unpublished data). In human research the moderator effect of familiarity on the androgen response to competition has seldom been considered, but in two studies with coalitional competition in domino (Wagner et al., 2002) and in video-game tournaments testosterone increased in response to out-group but not to in-group contests (Oxford et al., 2010). Other previous work has either ignored this variable or excluded participants with some degree of familiarity by asking contestants that knew each other to sign up for different experimental sessions (e.g., Mehta et al., 2009).

In this study we aim at investigating the effects of opponent familiarity and the evaluation of the competition outcome as threat or challenge on the T response to competition. We have also measured the levels of cortisol (C), since it is known to respond and interact with T when individuals are facing a social challenge (e.g., Viau, 2002; Mehta and Josephs, 2010), and of DHEA since it is the most prevalent androgen for women (Labrie, 2010) and is involved in the regulation of aggressive behavior (e.g., Soma et al., 2008).

## MATERIALS AND METHODS

### PARTICIPANTS AND EXPERIMENTAL PROTOCOL

Thirty-four undergraduate psychology female students (21.29 ± 3.41 years), signed up to participate in experimental sessions of approximately 1 h, scheduled to 12:30 and 17:30 to control for circadian variation of hormone levels. Participants were tested in pairs (17 dyads) and were rewarded with one course credit and 12 euros, depending of their competitive task outcome (winners: one course credit and 12 euros; losers: one course credit). All experimental sessions were conducted by a male and a female experimenter. This experiment was performed in accordance to national regulations and with the approval of the ethics committee of ISPA’s Research Centre. Written consent was given by all participants.

### DATA COLLECTION AND PSYCHOLOGICAL VARIABLES

Participants were asked to sit face to face across a table, in which a vertical barrier had been placed. This barrier allowed the participants to see their opponent, but restricted the view of the opposite side of the table in such a way that they were unable to see what the opponent was doing during all stages of the competition. Upon arrival the participants provided a baseline saliva sample and filled in the demographic questions, including use of oral contraceptives (OCs) and the date of the last menstruation. Pairs were asked to rate from 1 to 5 how familiar they were with each other prior to this experiment (1 = not familiar; 5 = very familiar). Familiarity was conceptualized as a continuous signal-detection process (e.g., Yonelinas, 1997) and therefore we have avoided a dichotomic classification of “familiar vs. unfamiliar” that would create artificial groups and would not reflect the nature of this variable. For the competitive task we have used the number tracking test (NTT) and this task was introduced to the competitors after completing the first set of questionnaires. The NTT has been used before in competition experiments (e.g., Schultheiss and Rohde, 2002; Carré et al., 2009) and requires participants to connect a sequence of consecutive ascending numbers (1-2-3-4-…) arranged in a matrix and surrounded by distracting numbers. Instructions focused on the competitive nature of the task, by stressing that participants will compete against one another for 12 Euros on a set of trials each associated with a specific NTT matrix. Feedback about who was the first to reach the highlighted end number on each NTT matrix characterized a trial as a “Win” or a “Loss” to the participant. Easy and difficult matrices were created by manipulating the distance between the start and the end number. This procedure allowed an undetectable experimental manipulation of the outcome (winning or losing the competition) and has been used in previous research (e.g., Schultheiss and Rohde, 2002; Wirth et al., 2006; Carré et al., 2009). Participants were also unaware of the relative difficulty of the matrices since they had no access to their opponent matrices.

Before the competitive NTT trials, participants were allowed to complete a NTT matrix for training purposes. For the competition the NTT was arranged in three sets of four NTT matrices. The first and second NTT sets were manipulated in such a way that the participants would have equal number of victories and defeats (four wins, four losses) before entering the third set. On the third NTT set, the participant in the winner condition would win the four NTT duels and the participant in the loser condition would lose the four NTT duels. The outcome of two pairs violated the expectation (i.e., participant in the winner treatment lost the competition). These participants were coded to their actual competition outcome and included in the sample (see Preliminary analysis for testing). It was tested if the removal of these participants from the sample would affect the results and it was found that the main results remain the same.

After the competition outcome was announced, payment was given to the participants according to their task outcome. At this point evaluation of the competition outcome was individually assessed by scoring it as a threat and as a challenge using two items with a four points scale (e.g., I consider my participation in this study as: 1 = not threatening; 4 = very threatening; I consider my participation in this study as: 1 = not challenging; 4 = very challenging) inspired by Tomaka et al. (1993,^1997^). Personality questionnaires unrelated to this experiment were then distributed to occupy the participants until the collection of a post-competition saliva sample 20 min after the end of the competition, which ended the experimental session (as in Oliveira et al., 2009).

### HORMONE ASSAYS

Saliva samples were collected on 5 ml polypropylene vials and stored at -20°C immediately after the end of the experimental session. Samples were thawed, centrifuged at 3600 r.p.m. (2245 × *g*) for 10 min and the supernatant stored at -20°C until the assay. Hormone assays were conducted using IBL (Hamburg, Germany) LIA kits for T, C and DHEA. The intra-assay and inter-assay coefficients of variance were respectively, 6.1 and 8.6% for T, 8.3 and 12.4% for C, and 4 and 11.9% for DHEA.

### PRELIMINARY ANALYSIS

All hormone values were log-transformed for statistical analysis due to skewness and violation of the parametric test assumptions (see **Table 1** for absolute values). This transformation is a common procedure for the analysis of hormonal data (e.g., Wirth et al., 2006; Mehta et al., 2009). All sampling points of the measured hormones were scanned for outliers (three standard deviations) and no participants were excluded based on this criterium. Degrees of freedom vary for the statistical analysis of DHEA, due to an insufficient volume of saliva to carry on the hormone assay for the baseline measurement of two participants. Participants were controlled for the phase of the menstrual cycle and for the use of OCs. Phase of the menstrual cycle was excluded from the analysis, since the number of participants in each category was insufficient for testing (number of winners per phase of the menstrual cycle: follicular = 2, ovulation = 1, luteal = 2; number of losers per phase of the menstrual cycle: follicular = 1, ovulation = 2, luteal = 4). Furthermore, previous research has failed to find an effect of menstrual cycle over the patterns of variation in T and C (e.g., Dabbs and de La Rue, 1991; Liening et al., 2010).

**Table 1:** Absolute values for all sampling points of the measured hormones.

T1 (pg/ml)		T2 (pg/ml)		C1 (ng/ml)		C2 (ng/ml)		DHEA1 (pg/ml)		DHEA2 (pg/ml)	
Mean	SEM	Mean	SEM	Mean	SEM	Mean	SEM	Mean	SEM	Mean	SEM
Winner	85.546	44.940	55.345	10.604	2.181	0.257	2.597	0.354	335.121	75.862	335.746	71.113
Loser	57.209	13.991	160.322	58.388	2.703	0.408	3.304	0.393	402.359	102.635	476.731	85.374

A repeated measures analysis of variance (ANOVA) was used to check for effects and interactions of OC on hormone levels. Previous research has shown that the use of OC does not affect the androgen response to competition (Edwards and O’Neal, 2009) and we have not found an effect of OC on hormones either for winners (T: Main effect: *F*(1, 15) = 1.637, *p* = 0.220, Interaction: *F*(1, 15) = 0.957, *p* = 0.343; C: Main effect: *F*(1, 15) = 0.700, *p* = 0.416, Interaction: *F*(1, 15) = 0.163, *p* = 0.691; DHEA: Main effect: *F*(1, 14) = 0.284, *p* = 0.602, Interaction: *F*(1, 14) = 1.050, *p* = .322) or for losers (T: Main effect: *F*(1, 14) = 0.040, *p* = 0.845, Interaction: *F*(1, 14) = 2.09, *p* = 0.170; C: Main effect: *F*(1, 14) = 1.470, *p* = 0.245, Interaction: *F*(1, 14) = 0.658, *p* = .430; DHEA: Main effect: *F*(1, 13) = 0.243, *p* = 0.630, Interaction: *F*(1, 13) = 0.286, *p* = 0.601), therefore this factor was also excluded from further testing. We have checked if the patterns of endocrine response for winners and losers were different when the competition outcome was the one predicted by the NTT matrices manipulation or not, and neither test reached statistical significance [T: *F*(1, 15) = 0.543, *p* = 0.472; C: *F*(1, 15) = 0.043, *p* = 0.837; DHEA: *F*(1, 13) = 0.092, *p* = 0.767]. Familiarity was measured but not manipulated. Familiarity ratings between participants ranged from 1 to 5 [mean = 3.13 ± 1.61].

### STATISTICAL ANALYSIS

We have used a mixed model analysis of covariance (ANCOVA) with outcome (winner, loser) as a within variable since we are comparing pairs of participants, familiarity as a covariate and each dependent variable as a repeated measures factor. Dependent variables that were tested in separate ANCOVA were: evaluation (threat, challenge), and the steroid hormones T, C, and DHEA (pre-, post-competition). All comparisons were performed using planned contrasts within the ANCOVA, therefore the degrees of freedom match those of the model.

Moderation analysis followed the procedure outlined by Aiken and West (1991). The unstandardized residuals scores from regressing the pre-competition T on post-competition T, were used as an index of T response (Allison, 1990; Mehta et al., 2008) and inserted as the dependent variable on the moderation model. Threat was centered and used as a predictor and familiarity was also centered and used as the candidate moderator. The interaction term was composed by the product of threat and familiarity. To control for abnormal contributions to the regression model from any individual observation, residuals were scanned for outliers (3 standard deviations). Using this criteria one case was excluded and the linear regression model was adjusted without the outlier observation. Simple slope tests for high and low levels of familiarity were also calculated as suggested by Aiken and West (1991). Similar moderation procedures have been used by Mehta et al. (2008) and van der Meij et al. (2010).

## RESULTS

### EVALUATION OF THE OUTCOME

#### Threat/challenge (**Figure 1**)

**FIGURE 1:**
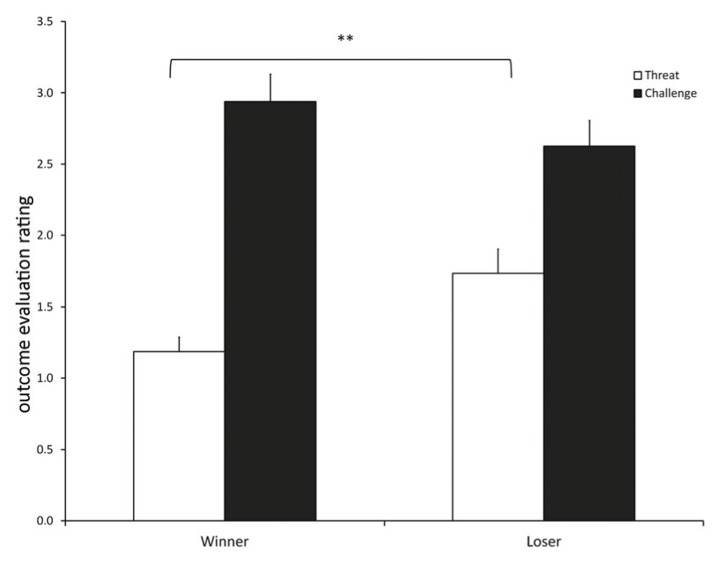
**Competition outcome evaluation rating as a threat/challenge (mean ± SEM) for participants in the winner and loser condition with familiarity of the opponent as a covariate.** (**) indicates significant differences at *p* ≤ 0.01.

The competition outcome was differently evaluated by winners and losers [*F*(1, 14) = 36.369, *p* < 0.001]. Participants in the loser condition evaluated the competition outcome as more threatening than winners [contrast: *t*(14) = 3.621, *p* = 0.002], while winners tended to evaluate the outcome more as a challenge than losers, although this difference was not significant [contrast: *t*(14) = 1.893, *p* = 0.079].

### HORMONAL VARIABLES

#### Testosterone (**Figure 2A**)

**FIGURE 2:**
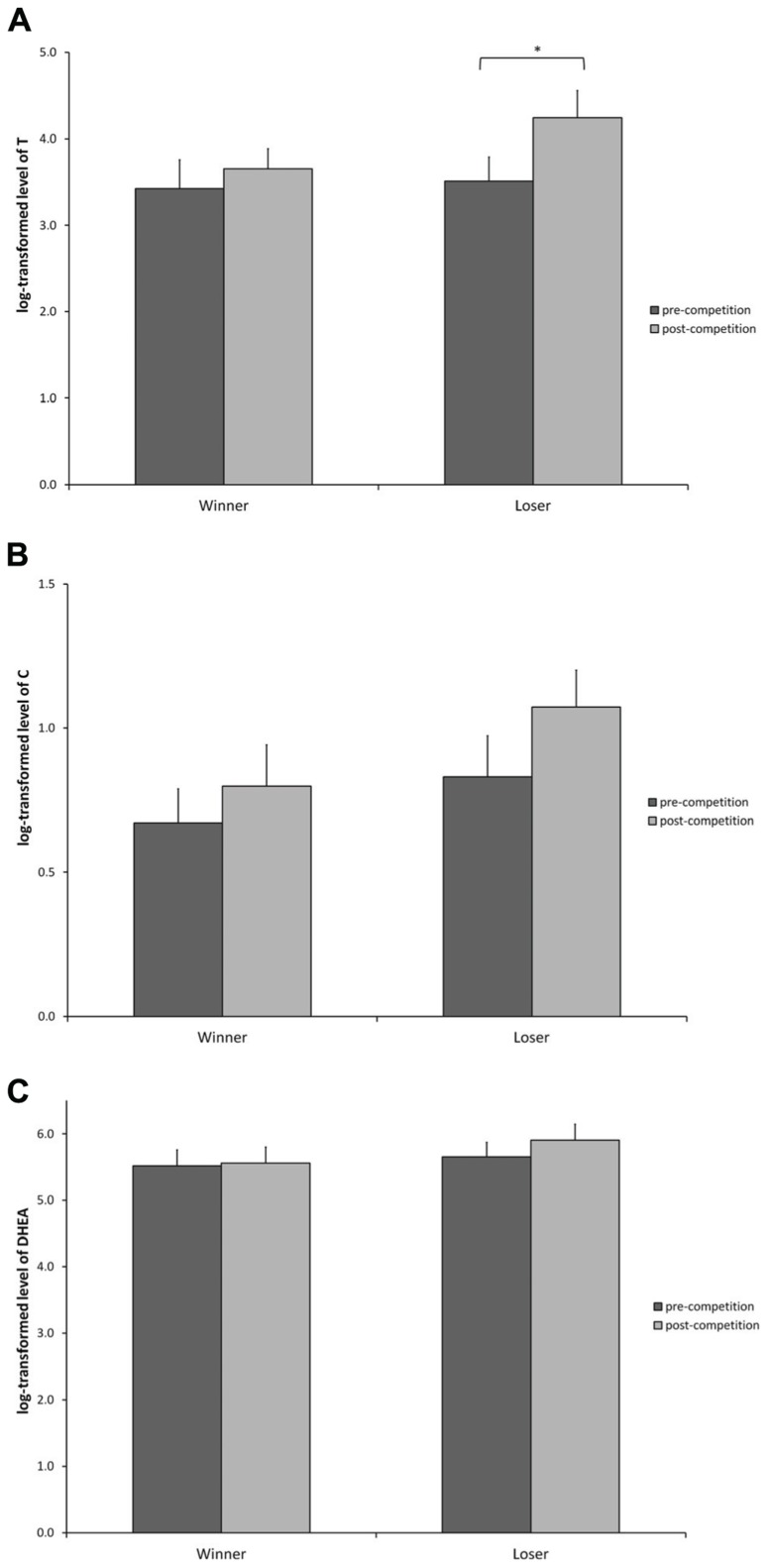
**log-transformed hormone levels (Mean ± SEM) measured at baseline level, and 20 min after the competition for participants in the winner and loser condition with familiarity of the opponent as a covariate.** **(A)** Testosterone, **(B)** cortisol, and **(C)** DHEA. (*) indicates significant differences at *p* ≤ 0.05.

A main effect of the competition outcome was found suggesting that losers have overall higher T than winners [*F*(1, 15) = 8.452, *p* = 0.010]. Subsequent contrast analysis showed that there were no baseline differences in T levels between winners and losers [contrast: *t*(15) = 0.186, *p* = 0.854] and that only losers significantly increased their levels of T from pre- to post-competition [contrast: *t*(15) = 2.488, *p* = 0.025]. The difference between the winner and loser condition after the competition did not reach statistical significance [contrast: *t*(15) = 1.769 *p* = 0.097].

#### Cortisol (**Figure 2B**)

Statistical analysis for C suggests that there was no overall variation of C levels throughout the competition [*F*(1, 15) = 1.035, *p* = 0.325] and that C levels were not different in both experimental conditions [*F*(1, 15) = 1.970, *p* = 0.180].

#### DHEA (**Figure 2C**)

A non-significant trend was found for DHEA levels to be higher at the end of the competition [*F*(1, 13) = 3.317, *p* = 0.091]. DHEA levels were not different between winners and losers neither at the baseline nor at the post-competition measure [Winner contrast: *t*(13) = 0.613, *p* = 0.550; Loser contrast: *t*(13) = 1.300, *p* = 0.216], but losers showed a non-significant trend to have higher DHEA after the competition [*t*(13) = 1.845, *p* = 0.088]. Winners show no changes in DHEA levels from pre- to post-competition [*t*(13) = 0.326, *p* = 0.749].

### ASSOCIATION BETWEEN HORMONES AND PSYCHOLOGICAL VARIABLES

No association was found between the ratings of the competition as challenge and any of the measured hormones for winners (all *p* > 0.292) and losers (all *p* > 0.641). Familiarity, Threat and T were only significantly correlated in the loser condition (see **Table 2**). Post-competitive levels of C and DHEA did not correlate either with threat or with familiarity.

**Table 2:** Pearson correlation coefficients between threat, familiarity and hormone levels 20 min after the competition for winners (n = 17) and losers (n = 17).

	Threat	Familiarity	T2	C2	DHEA2
Winner	Threat	1	0.359	0.073	-0.037	0.256
	Familiarity	0.359	1	0.424	0.054	0.235
Loser	Threat	1	-0.541^*^	0.630^**^	0.101	0.338
	Familiarity	-0.541^*^	1	-0.506^*^	-0.462	-0.218

* Significant for *p* < 0.05.

** Significant for *p* < 0.01.

### MODERATION ANALYSIS OF THREAT PERCEPTION AND FAMILIARITY ON T LEVELS FOR THE LOSER CONDITION

T response for participants in the loser condition was calculated as the unstandardized residuals of regressing baseline logT on logT 20 min after the competition (*R*^2^ = 0.496, *p* = 0.002).

The regression equation used to test the moderation effect with T response as the dependent variable, threat as predictor, familiarity as the moderator and the interaction between threat and familiarity was significant (*R*^2^ = 0.762, *p* < 0.001). The predictor Threat (β = 0.278, *p* = 0.149) and familiarity (β = -0.287, *p* = 0.114) were not significant, however, the interaction term threat × familiarity was highly significant (β = -0.613, *p* = 0.002). The inclusion of the interaction term also increased the explained variance of the regression model (Δ*R*^2^ = 0.317, *p* = 0.002).

**FIGURE 3:**
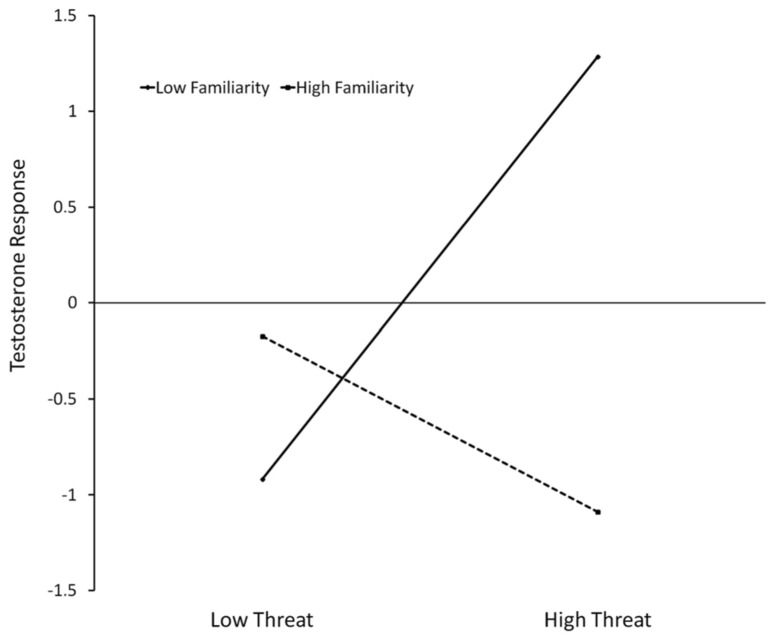
**Regression slopes predicting testosterone response (unstandardized residuals) in function of threat and familiarity for women that lost the competition.** High familiarity = 1 SD above mean, low familiarity = 1 SD below mean. Low threat = minimum observed value, high threat = maximum observed value.

Since the interaction of threat × familiarity was significant, we have conducted simple slopes analysis (Aiken and West, 1991; Mehta et al., 2008) for the relationship between T changes and Threat, one standard deviation above and below the mean of familiarity. Slope testing (**Figure 3**) shows that when the opponent is not familiar, higher threat leads to increases of T (*b* = 1.102, *t*(12) = 4.935, *p* = 0.0003), but no significant effect was found for familiar opponents (*b* = -0.458, *t*(12) = 1.360, *p* = 0.198).

## DISCUSSION

In this experiment we investigated how opponent familiarity and evaluation of the competition outcome could modulate the hormonal response to competition.

Contrary to the predictions of the challenge hypothesis (Wingfield et al., 1990) and previous findings in our lab (Oliveira et al., 2009) we did not find a clear winning/losing effect with higher post-competition T levels for winners and a decrease in T levels in losers. In fact, a significant hormonal response could only be found for the participants that were assigned to the loser condition. This group responded with increased post-competitive T levels, whereas the post-competition levels of the other measured hormones (C and DHEA) did not differ from pre-competitive values.

It could be argued that the rising T levels in losers but not in winners could be stress related in losers. Indeed ovaries and adrenals produce approximately the same percentage (25%) of circulating T in women (Burger, 2002), and adrenal androgens respond to stress (e.g., Oberbeck et al., 1998). However, in this study neither C nor DHEA, both of adrenal origin, were found to have a similar response to that of T, and previous studies have reported opposite effects of competition on T and C levels (Jiménez et al., 2012), which together suggest an independent response of the hormones to competition. Moreover, simulated competitive team matches failed to increase T levels (Filaire and Lac, 2000), whereas real matches activate a T response in women, thus suggesting that it is the meaning of the competition that triggers the response rather than the physical stress involved in the competition (Edwards and O’Neal, 2009).

It was also in the loser condition that the highest threat ratings were found and for which there was an association between post-competitive T, threat and opponent familiarity. As the moderation analysis has shown, the significant changes in T levels that were detected in this group in response to competition resulted from a moderator effect of the familiarity with the opponent on the evaluation of the outcome as a threat. When these participants lost the competition against an unfamiliar opponent, T levels increased when the evaluation of the task as threat was high. If the competition was lost against a familiar opponent, variations of threat intensity did not lead to changes in T levels.

These findings are congruent with evidence from non-human experiments in which familiarity with the opponent moderates the level of elicited aggression as a function of the threat imposed by the opponent (e.g., less aggression elicited by neighbors than by strangers in territorial systems where neighbors, that are also territory owners, impose a lower threat than floaters that are looking for territory take-overs, Ydenberg et al., 1988; Temeles, 1994). Accordingly, a recent study in our lab using a cichlid fish also found that the androgen response to a territory intrusion in a cichlid fish was moderated by the familiarity with the intruder (R. F. Oliveira, R. Aires, T. Oliveira, and A. Ros, unpublished data).

The link between higher threat and losing the competition is also coherent with appraisal theory. A threat evaluation may occur when the demands exceed the resources mobilized by the individual to respond to a social challenge (Blascovich and Mendes, 2000). Since the competition outcome was experimentally manipulated, if the participants are motivated and engage in competition a higher threat evaluation is to be expected in the loser condition where participants will always perceive their resources to be insufficient to reverse the score and win the competition. Likewise, it would be possible that the task outcome exerted a suppressing effect over the threat evaluation of the competition for participants in the winner condition, as the resources possessed by the individual were sufficient to resolve the interaction in their favor (Blascovich and Mendes, 2000). In this context the lack of T response in winners can be seen has having an economical and adaptive value, while an increase in losers can be interpreted as a physiological response that prepares the individual for future encounters to regain lost status or to buffer the individual in case of an extended contest (Schultheiss et al., 2005; Mehta and Josephs, 2006). Interestingly the effects of T administration on relevant psychological processes (e.g., perception of threatening faces) for future competition are also moderated by contextual and personal factors (see review by Bos et al., 2012).

The hypothesis that the endocrine response to competition is triggered by the individuals’ evaluation instead of by the objective structure of the competitive task is a possible explanation for the divergences in T response patterns to competition (Oliveira et al. , 2005; Salvador, 2005; Salvador and Costa, 2009). In fact, the range of reported androgen responses to competition in the literature varies from T increases in winners, no significant response or even T increases in losers (e.g., van Anders and Watson, 2007; Hamilton et al., 2009; Salvador and Costa, 2009). In this respect, the evaluation of threat/challenge (*sensu *Blascovich and Mendes, 2000) posed by the competition outcome is a good candidate for moderating the T response.

In summary, the results presented here support the view that the subjects’ evaluation of the event plays a key role in the activation of a T response to competition in women and could partly account for intersexual differences in the endocrine response to competition (e.g., Salvador and Costa, 2009), illustrating the need for further studies in which the moderator role of different appraisal dimensions of the competitive event on hormonal responses to competition is formally tested.

## Conflict of Interest Statement

The authors declare that the research was conducted in the absence of any commercial or financial relationships that could be construed as a potential conflict of interest.
